# Heated tobacco product use frequency, smoking quit attempts, and smoking reduction among Mexican adult smokers

**DOI:** 10.18332/tid/187576

**Published:** 2024-05-29

**Authors:** Lizeth Cruz-Jiménez, Inti Barrientos-Gutiérrez, Dèsirée Vidaña-Pérez, Katia Gallegos-Carrillo, Edna Arillo-Santillán, Rosibel Rodríguez-Bolaños, James W. Hardin, Minji Kim, James F. Thrasher

**Affiliations:** 1 Department of Health Promotion, Education and Behavior, Arnold School of Public Health, University of South Carolina, Columbia, United States; 2 Evaluation and Survey Research Center, National Institute of Public Health, Cuernavaca, Mexico; 3 Epidemiology and Health Services Research Unit, Mexican Institute of Social Security, Cuernavaca, Mexico; 4 Tobacco Research Department, National Institute of Public Health, Cuernavaca, Mexico; 5 Reproductive Health Department, National Institute of Public Health, Cuernavaca, Mexico; 6 Department of Epidemiology and Biostatistics, Arnold School of Public Health, University of South Carolina, Columbia, United States

**Keywords:** IQOS, cessation behaviors, novel tobacco products, e-cigarette users

## Abstract

**INTRODUCTION:**

Heated tobacco products (HTPs) are promoted as less harmful than cigarettes; nonetheless, whether HTPs help smokers quit is uncertain.

**METHODS:**

Data from 4067 Mexican adult smokers surveyed longitudinally every four months (November 2019–March 2021) were analyzed. Mixed-effects multinomial models regressed HTP use frequency (no use=reference; monthly; weekly; and daily use) on sociodemographics and tobacco/nicotine-related variables. Among participants who completed at least two surveys (n=2900) over four months, the duration of their longest smoking quit attempt (SQA) between surveys (SQAs: <30 days; ≥30 days; no SQA=reference) was regressed on HTP use frequency, and changes in the number of cigarettes smoked per day were regressed on HTP initiation between surveys, adjusting for covariates.

**RESULTS:**

Consistent predictors of all HTP use frequencies (monthly, weekly, or daily vs no use) were daily smoking >5 cigarettes (ARRR=1.69 [95% CI: 1.12–2.55], 1.88 [95% CI: 1.26–2.81] and 6.46 [95% CI: 3.33–12.52], respectively); e-cigarette use (ARRR =5.68 [95% CI: 3.38–9.53], 6.54 [95% CI: 4.06–10.55] and 2.59 [95% CI: 1.26–5.30]); lower HTP risk perceptions (ARRR=2.12 [95% CI: 1.50–30.00], 2.25 [95% CI: 1.63–3.10] and 2.00 [95% CI: 1.25–3.22]); exposure to HTP information inside (ARRR=2.13 [95% CI: 1.44–3.15], 2.13 [95% CI: 1.49–3.05] and 3.72 [95% CI: 2.28–6.09]) and outside stores (ARRR=2.36 [95% CI: 1.56–3.57], 2.32 [95% CI: 1.65–3.25] and 2.44 [95% CI: 1.41–4.24]) where tobacco is sold; having family (ARRR=2.46 [95% CI: 1.54–3.91], 2.90 [95% CI: 1.93–4.37] and 2.96 [95% CI: 1.52–5.77]) and friends (ARRR=5.78 [95% CI: 3.60–9.30], 4.98 [95% CI: 3.22–7.72] and 6.61 [95% CI: 2.91–15.01]) who use HTPs. HTP use frequency was not associated with quit attempts, except for monthly HTP use predicting SQAs lasting ≥30 days (ARRR=2.12 [95% CI: 1.17–3.85]). Initiation of HTP use was not associated with changes in smoking frequency. Limiting analysis to those who intend to quit smoking also yielded null results.

**CONCLUSIONS:**

Among Mexican adult smokers, frequency of HTP use was mostly not associated with either cessation behaviors or changes in cigarette consumption, suggesting that HTPs have limited to no effectiveness for smoking cessation.

## INTRODUCTION

Heated tobacco products (HTPs) have gained popularity since their introduction about a decade ago^1^. Although evidence of the health effects of HTPs use is inconclusive^2^, the tobacco industry promotes HTPs as a less harmful alternative to combustible cigarettes^3^. Whether HTPs help smokers quit remains unknown, and there is scarce research differentiating among those who use HTPs more or less frequently. This study assesses the correlates of HTP use frequency, whether HTP use predicts smoking quit attempts (SQAs), and whether HTP initiation is associated with a change in smoking frequency among Mexican adult smokers.

The HTP market growth has been the strongest in five countries – Japan, Korea, Russia, Italy, and Germany – that accounted for over 50% of the global HTP market share in 2020^4^. Japan and Korea, in particular, witnessed strong market growth for these novel tobacco products, even when compared to e-cigarettes^5^. IQOS, by Philip Morris International (PMI), stands as a market leader within these regions while having a more modest presence in Latin America. In Latin America, HTPs have exhibited a consistent growth trajectory since 2017, and continued growth is expected^4^.

The increased popularity of HTPs has been accompanied by the industry’s often misleading marketing strategies^6^. Marketing materials often claim HTPs are ‘less harmful’ than traditional cigarettes in spite of the lack of conclusive evidence that they actually reduce harm overall^7,8^. In 2020, the United States (US) Food and Drug Administration (FDA) authorized IQOS to be marketed as a modified-risk tobacco product based on the assumption that smokers who completely switch to HTPs will reduce their exposure to harmful chemicals, though the FDA did not allow the use of reduced risk claims^9^. Nevertheless, PMI highlighted the FDA’s ruling on IQOS in its promotional materials and website, omitting the FDA’s denial of reduced-risk claims and other relevant findings about the risks and characteristics of IQOS^8^.These actions may have perpetuated misperceptions about HTPs reducing harm from smoking^3^.

Claims about reduced risks from HTP use hinge on their utility for stopping smoking, yet no independent research has supported industry claims that HTPs promote smoking cessation. A study of Korean cigarette smokers found that those who used HTPs were no more likely to quit smoking than those who only used combustible cigarettes, regardless of their cigarette smoking frequency^10^. Furthermore, a representative cohort study in Japan found that after one year of follow-up, HTP users were less likely to achieve at least one month of abstinence compared to established cigarette smokers who had used evidence-based cessation measures. In the same study, among people who had quit smoking for more than one year, HTP use was associated with a higher risk of smoking relapse^11^. These findings are consistent with a randomized clinical trial in Hong Kong, where HTP use was not associated with cigarette abstinence at six months of follow-up among adult smokers who want to quit or reduce smoking^12^. Despite this evidence, the industry still claims HTPs are a better alternative for those who are concerned about the consequences of smoking, which promotes switching rather than complete quitting, and have advocated for their product to be sold in other markets.

Regulation of HTPs varies widely between countries, which can impact their development and effect^13^. In emerging markets, such as Latin America, there is a need to understand whether HTPs are replacing combustible cigarettes. In 2018 in Mexico, PMI started to promote IQOS through social media campaigns to advance its introduction in physical retail stores by the end of 2019. The import and export of HTPs were banned in early 2022 in Mexico, explicitly prohibiting their sale and distribution^14^. Nevertheless, specialized IQOS stores that were established prior to the prohibition persist as the sole brand on the market, taking advantage of a regulatory loophole. Two recent studies among Mexican smokers suggested that despite great interest in trying HTPs (75%)^15^, the prevalence of HTP use was low (1.1%) following their introduction in 2019, with current e-cigarette use being a particularly strong correlate for HTP use^16^. There is a crucial need for evidence to guide regulation development that considers their potential public health impact. Thus, this exploratory study assesses the correlates of HTP use frequency among adult smokers and whether HTP use predicts smoking cessation behaviors and changes in smoking frequency.

## METHODS

### Data source

Data used were from five waves (November 2019 – March 2021) of an open cohort of Mexican smokers and e-cigarette users who were surveyed every four months through a non-probability sample of participants from an online consumer panel. People who participated in the study were aged ≥18 years and reported smoking or using e-cigarettes within the last 30 days (i.e. current users). To ensure a consistent sample size of about 1500 participants at each wave, the sample was replenished to account for loss to follow-up, with the original objective of evaluating transitions in e-cigarette use. Quotas were used for e-cigarette use in the last month (n>500) and education level (>500 with high school or less).

**Figure 1:**
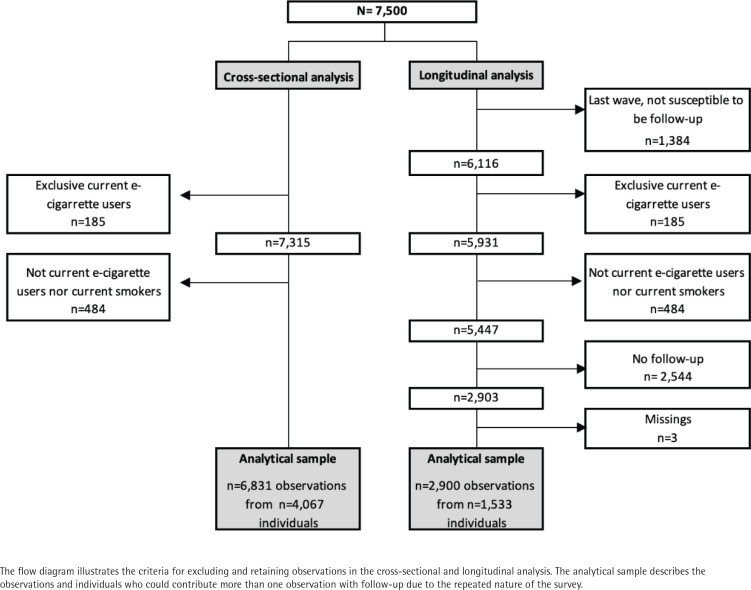
Study flow diagram

The analytic sample for this study included 6831 observations from 4067 participants who were current cigarette smokers at the time of the survey (Nov. 2019, n=1389; Mar. 2020, n=1351; July 2020, n=1330; Nov. 2020, n=1377; and Mar. 2021, n=1384), of whom 2318 observations corresponded to 1496 individuals who also used e-cigarettes (i.e. dual users). Exclusive e-cigarette users were dropped due to the small sample size (i.e. 185 observations from 166 individuals) (Figure 1). For evaluating quit attempts and changes in smoking frequency, a second analytic sample including only participants with at least one 4-month follow-up survey was used (2900 observations from 1533 individuals) (Figure 1). Surveys were self-administered online in Spanish using standard questions on tobacco product use and questions on novel tobacco products from the International Tobacco Control (ITC) survey^17^. Participants provided consent before completing the survey, which took a median time of about 23 minutes to complete, and they received standard compensation for online panelists (i.e. points for e-gift certificates). All procedures were approved by the Institutional Review Board and Ethics Committee of the Mexican National Institute of Public Health (Ethical Approval Code: CI 1572).

### Primary dependent variables

*Smoking quit attempts (SQAs)*

Participants who answered two consecutive surveys reported at ‘t+1’ (i.e. four months after the prior survey) if they had tried to quit smoking in the prior four months and, if so, the duration of the longest quit attempt during that period. We combined responses to these questions to derive a variable for SQAs at ‘t+1’ (i.e. no SQA; SQA<30 days; SQA≥30 days).

*Change in cigarette smoking frequency*

Changes in smoking frequency were estimated by subtracting the self-reported number of cigarettes smoked per day at ‘t+1’ from that reported in the previous survey, assigning 0 to those who had quit smoking at follow-up. As non-daily smokers were asked the average number of cigarettes smoked per week, this number was divided by seven for the consistent daily total. Thus, a positive change represented an increase in the average number of cigarettes smoked each day, and a negative change represented a decrease.

### Primary independent variables

*HTP use*

Before a series of questions about HTPs, participants were shown an image of the PMI IQOS (heat stick, holder, charger) with a brief description emphasizing how IQOS differs from cigarettes or e-cigarettes. An image of IQOS was chosen due to its exclusivity as the sole brand available within this context. Participants then self-reported their awareness of HTPs (i.e. yes, no, don’t know), ever trial of HTPs (i.e. yes, no, don’t know), and frequency of HTP use in the last 30 days (i.e. not at all; less than once a month, but occasionally; less than weekly, but at least once a month; less than daily, but at least once a week; daily). The answers to these inquiries were utilized to create a 4-level variable that reflects HTPs use frequency in the prior 30 days: no current use (i.e. unaware of HTPs or no HTP use in last month); use <once/week, but at least once a month; ≥once/week, but not daily; and daily use). Among participants who answered two consecutive surveys within four months (i.e. at time ‘t’ and time ‘t+1’), we used the same questions to derive a variable to reflect changes in HTP use between consecutive surveys (i.e. time ‘t’ and time ‘t+1’): never HTP user (i.e. non-user at t and t+1); HTP quitter (user at t, non-user at t+1); persistent HTP user (i.e. user at t and t+1); and new HTP user (non-user at t, user at t+1).

Finally, intention to quit smoking within the next six months (yes vs no) was considered to derive four groups based on current HTPs use at time t: intention to quit smoking, no HTP use= reference group; intention to quit smoking, current HTP use; no smoking quit intention or HTP use; and no smoking quit intention, current HTP use.

### Other variables related to tobacco products

Participants were asked about their perception of HTPs’ harmfulness relative to cigarettes, with responses recoded to indicate lower risk of HTPs (yes vs equal or higher risk, ‘don’t know’, or unaware of HTPs). Participants reported their exposure to information related to HTP in the last 30 days for three locations: inside shops/stores that sell tobacco products, outside shops/stores that sell tobacco products, and in newspapers or magazines. Each response option was dichotomized (yes vs no), and respondents who were unaware of HTPs were coded as ‘no’.

Participants’ smoking frequencies were categorized using cutoff points that reflected membership in tertiles of consumption intensity among Mexican smokers (non-daily; daily ≤5 cigarettes, and daily >5 cigarettes) who have a relatively light smoking pattern^18,19^. Participants were queried about their e-cigarette use in the prior month (yes vs no). Individuals also reported whether they had tried to quit smoking in the prior four months (yes vs no) and their intentions to quit smoking in the next six months (yes vs no).

At all but the last survey wave, nicotine dependence was evaluated using 10 items from the Wisconsin Inventory of Smoking Dependence Motives (WISDM). Their selection was based on data regarding their utility among Mexican Americans, who, like Mexicans, typically exhibit a low frequency smoking pattern, with items drawn from various subscale domains (i.e. automaticity, cravings, cue exposure-associative processes, negative reinforcement, positive reinforcement, weight control). Reliability is high (Cronbach’s alpha = 0.92), and responses were averaged.

Participants indicated whether they had a partner or spouse who smokes and, in a separate question, whether any other family member smokes. Responses were aggregated to indicate smoking in the household, distinguishing between those with and without a smoking partner or family member (yes vs no). We assessed e-cigarette use among partners and family members in the same manner. Participants also were queried about HTP use among any household family members (yes vs no). Current smoking, e-cigarette use, and HTP use among the five closest friends of the participants were assessed by separate questions for each product (recoded to yes vs no). For these variables, participants who responded ‘don’t know’ or who skipped the HTP questions because they were unaware of HTPs, were coded as ‘no’.

### Other covariates

Sociodemographic characteristics queried included sex (male, female), age (18–29, 30–39, 40–49, and ≥50 years), education level (university or higher; technical and some college; and high school or lower), and monthly household income denoted in MXM (1000 Mexican pesos about US$59): <8000, 8001–15000, ≥15001, and ‘don’t know’. Other covariates included survey wave (i.e. Nov. 2019, Mar. 2020, July 2020, Nov. 2020, and Mar. 2021) and a time-in-sample variable (i.e. from 1 to 4) to indicate the number of prior surveys to which participants had responded at time t.

### Analysis

The entire sample was used to estimate crude and adjusted relative risk ratio (RRR and ARRR, respectively) of HTP use frequency (i.e. less than once a week, but at least once a month; not daily, but at least once a week; daily use; and no current HTPs use = reference), using mixed-effects multinomial logistic regression with random effects and exchangeable covariance matrix structure to account for repeated measures. Analyses were adjusted by time-in-sample and covariates (i.e. sociodemographics, smoking-related variables, with the exception of dependence, HTPs relative risk perception, HTPs information exposure and smoking, e-cigarette use, and HTPs use behaviors among family and friends). To maximize the sample size, we report results without nicotine dependence (not assessed at the final survey); results from sensitivity analyses on the smaller sample that included dependence were consistent (Supplementary file Table 1).

Longitudinal analyses evaluated only participants with follow-up of at least two consecutive surveys (i.e. t+1). To predict SQAs at a time t+1 (i.e. no SQA=reference; SQA<30 days; SQA≥30 days), mixed-effects multinomial logistic regression models with random effects evaluated HTP use frequency at a time t and, in a separate model, intention to quit smoking (yes, no) by HTPs use (yes, no) at time t (reference group=intention to quit smoking, no HTP use).

Finally, we estimated two generalized estimating equations (GEE) linear regression models with robust (to misspecification of the within-person correlation of responses) standard errors to predict changes in the number of cigarettes between surveys based on HTPs use from time t to t+1 (i.e. non-user at t and t+1=reference group; HTP user at t, non-user at t+1; persistent HTP user at t and t+1; non-user in t, user in t+1) and, separately, the intersection between smoking quit intention (yes, no) and current HTPs use (yes, no) at time t. All analyses were adjusted by time sample and covariates (i.e. sociodemographics, smoking-related variables, HTPs relative risk perception, HTPs information exposure and smoking, e-cigarette use, and HTPs use behaviors among family and friends). All tests were considered two-tailed and reported with a 95% CI. All analyses were conducted using Stata v.18 (Stata Corp, TX, USA).

## RESULTS

Sample characteristics are shown in Table 1. About a third (33.9%) of the sample used e-cigarettes (i.e. dual users), and 9.9% used HTPs in the prior 30 days. Approximately half of the participants were male (51.8%), with around a third were aged 30–39 years (30.1%). Additionally, a significant portion of participants had a high school education or lower (36.1%).

**Table 1:** Sample characteristics of smokers from an open cohort study in Mexico, 2019–2021*

*Characteristics*	*n*	*%*
**Sex**		
Female	3291	48.2
Male	3540	51.8
**Age** (years)		
18–29	1843	27.0
30–39	2057	30.1
40–49	1334	19.5
≥50	1597	23.4
**Education level**		
High school or lower	2468	36.1
Technical and some college	2074	30.4
University or higher	2289	33.5
**Household income** (MXN)		
<8000	1551	22.7
8001–15000	2068	30.3
≥15001	2875	42.1
I don’t know	337	4.9
**Smoking frequency** (cigarettes/day)		
Non-daily	3500	51.2
≤5	1548	22.7
>5	1783	26.1
**E-cigarette use**		
No	4513	66.1
Yes	2318	33.9
**Recent smoking quit attempt**		
No	4100	60.0
Yes	2731	40.0
**Intention to quit smoking** (next 6 months)		
No	4386	64.2
Yes	2445	35.8
**Current use of HTPs**		
No	6158	90.2
Yes	673	9.9
**Low relative risk perception of HTPs compared to combustible cigarettes**		
No	1444	21.1
Yes	1137	16.6
I don’t know	336	4.9
Unaware	3914	57.3
**Self-reported exposure to HTP information**		
Inside shops/stores that sell tobacco		
No	5954	87.2
Yes	877	12.8
Outside shops/stores that sell tobacco		
No	5635	82.5
Yes	1196	17.5
In newspapers or magazines		
No	5732	83.9
Yes	1099	16.1
**Partner/family smokes**		
No	2364	34.6
Yes	4467	65.4
**Partner/family use e-cigarettes**		
No	5195	76.1
Yes	1636	24.0
**Family use HTPs**		
No	5951	87.1
Yes	880	12.9
**Friends smoke**		
No	1250	18.3
Yes	5581	81.7
**Friends use e-cigarettes**		
No	4379	64.1
Yes	2452	35.9
**Friends use HTPs**		
No	5772	84.5
Yes	1059	15.5
**Survey**		
November 2019	1389	20.3
March 2020	1351	19.8
July 2020	1330	19.5
November 2020	1377	20.2
March 2021	1384	20.3

* The sample included 6831 observations from 4067 individuals who could contribute more than one observation with follow-up due to the repeated nature of the survey. MXN: 1000 Mexican pesos about US$59.

### Correlates of HTPs use frequency

Results (Table 2) indicated a higher likelihood of current monthly HTP use (i.e. less than once a week but at least once a month vs no current HTPs use) among people who: smoke >5 cigarettes daily (ARRR=1.69); used both cigarettes and e-cigarettes (ARRR=5.68); had a recent quit attempt (ARRR=1.53); perceived HTPs as less harmful than cigarettes (ARRR= 2.12); noticed information about HTPs inside and outside stores where tobacco products are sold (ARRR=2.13 and ARRR=2.36, respectively); had family members (ARRR=2.46) or friends (ARRR=5.78) who used HTPs. People were less likely to use HTPs monthly (vs no current HTPs use) if they were older (ARRR_40–49 vs 18–29_ = 0.54) and had family members who smoke (ARRR= 0.60).

**Table 2:** Mixed effects multinomial logistic regression^†^ of factors associated with HTPs use frequency among smokers from an open cohort study in Mexico, 2019–2021*

	*Do not currently use (N=6158)*	*Less than once a week, but at least once a month (N=235)*					*Not daily but at least once a week (N=338)*					*Daily (N=100)*				
	*%*	*%*	*RRR*	*95% CI*	*ARRR§*	*95% CI*	*%*	*RRR*	*95% CI*	*ARRR§*	*95% CI*	*%*	*RRR*	*95% CI*	*ARRR§*	*95% CI*
**Sex**																
Female ®	91.2	3.1	1		1		4.4	1		1		1.2	1		1	
Male	89.2	3.7	1.22	0.9–1.66	1.26	0.89–1.77	5.4	1.25	0.92–1.69	1.20	0.84–1.70	1.7	1.43	0.88–2.31	1.25	0.74–2.14
**Age**																
18–29 ®	88.6	4.6	1		1		5.3	1		1		1.5	1		1	
30–39	86.6	4.5	0.99	0.69–1.42	0.74	0.49–1.13	6.5	1.25	0.88–1.78	0.85	0.54–1.32	2.4	1.64	0.94–2.85	0.94	0.50–1.75
40–49	91.7	2.5	**0.52** ^b^	0.33–0.82	**0.54** ^a^	0.32–0.92	4.8	0.87	0.55–1.38	0.89	0.51–1.55	1.1	0.67	0.33–1.35	0.61	0.27–1.35
≥50	95.3	1.6	**0.32** ^c^	0.19–0.52	0.62	0.36–1.08	2.6	**0.46** ^b^	0.28–0.74	1.07	0.59–1.91	0.5	**0.31** ^b^	0.13–0.74	0.62	0.23–1.64
**Education level**																
High school or lower	93.4	2.4	**0.38** ^c^	0.27–0.53	0.71	0.46-1.07	3.5	**0.33** ^c^	0.24–0.46	0.69	0.45–1.06	0.7	**0.23** ^c^	0.12–0.46	0.48	0.21–1.07
Technical and some college	94.8	2.4	**0.37** ^c^	0.26–0.53	0.86	0.56–1.31	1.9	**0.18** ^c^	0.12–0.26	**0.49** ^b^	0.31–0.76	0.9	**0.27** ^c^	0.15–0.48	0.86	0.45–1.63
University or higher ®	82.4	5.6	1		1		9.3	1		1		2.8	1		1	
**Household income** (MXN)																
<8000 ®	93.8	93.8	1		1		2.7	1		1		1.0	1		1	
8001–15000	91.1	91.1	**1.52** ^a^	1.01–2.29	1.13	0.70–1.81	4.3	**1.64** ^a^	1.07–2.51	1.17	0.70–1.94	1.0	0.97	0.52–1.8	0.55	0.25–1.21
≥15001	86.6	86.6	**1.83** ^b^	1.24–2.71	1.16	0.72–1.88	7.0	**2.81** ^c^	1.88–4.20	1.53	0.89–2.63	2.2	**2.30** ^b^	1.28–4.15	1.03	0.48–2.21
I don’t know	97.3	97.3	0.35	0.11–1.11	1.21	0.41–3.55	1.5	0.53	0.20–1.37	2.08	0.80–5.41	0.3	0.28	0.04–2.09	1.16	0.10–13.68
**Smoking frequency** (cigarettes/day)																
Non-daily ®	93.4	2.9	1		1		3.3	1		1		0.4	1		1	
≤5	88.5	3.4	1.26	0.85–1.87	1.35	0.87–2.09	6.0	**1.90** ^c^	1.35–2.65	**1.96** ^c^	1.31–2.94	2.1	**5.09** ^c^	2.53–10.24	**5.53** ^c^	2.63–11.60
>5	85.3	4.6	**1.76** ^c^	1.26–2.46	**1.69** ^a^	1.12–2.55	7.2	**2.35** ^c^	1.69–3.27	**1.88** ^b^	1.26–2.81	3.0	**7.60** ^c^	4.15–13.92	**6.46** ^c^	3.33–12.52
**E-cigarette use**																
No ®	98.2	0.7	1		1		0.7	1		1		0.4	1		1	
Yes	74.5	8.8	**16.29^c^**	10.87–24.4	**5.68^c^**	3.38–9.53	13.2	**24.55^c^**	16.68–36.15	**6.54^c^**	4.06–10.55	3.6	**12.54^c^**	7.2–21.82	**2.59^b^**	1.26–5.30
**Recent smoking quit attempt**																
No ®	92.0	2.8	1		1		3.9	1		1		1.3	1		1	
Yes	87.4	4.4	**1.68^c^**	1.26–2.23	**1.53^a^**	1.05–2.23	6.5	**1.76^c^**	1.34–2.30	1.21	0.86–1.70	1.7	1.35	0.87–2.08	0.99	0.52–1.89
**Intention to quit smoking**																
No ®	91.1	3.4	1		1		4.2	1		1		1.3	1		1	
In the next six months	88.4	3.5	1.07	0.8–1.42	0.75	0.52–1.10	6.3	**1.53^c^**	1.18–1.98	1.29	0.92–1.81	1.8	1.45	0.94–2.23	1.41	0.78–2.56
**Low relative risk perception of HTPs compared to combustible cigarettes**																
No ®	94.4	2.0	1		1		2.8	1		1		0.8	1		1	
Yes	69.0	10.5	**7.03^c^**	5.26–9.40	**2.12^c^**	1.50–30.00	15.9	**7.90^c^**	6.10–10.24	**2.25^c^**	1.63–3.10	4.7	**7.73^c^**	5.15–11.59	**2.00^b^**	1.25–3.22
**Inside shops/stores that sell tobacco**																
No ®	94.9	2.0	1		1		2.6	1		1		0.5	1		1	
Yes	57.8	13.5	**11.24^c^**	8.36–15.1	**2.13^c^**	1.44–3.15	20.8	**13.00^c^**	10.08–16.78	**2.13^c^**	1.49–3.05	8.0	**26.01^c^**	16.15–41.88	**3.72^c^**	2.28–6.09
**Outside shops/stores that sell tobacco**																
No ®	95.8	1.6	1		1		2.1	1		1		0.5	1		1	
Yes	63.4	12.2	**11.69^c^**	8.52–16.04	**2.36^c^**	1.56–3.57	18.4	**13.28^c^**	10.30–17.13	**2.32^c^**	1.65–3.25	6.0	**18.32^c^**	11.00–30.49	**2.44^b^**	1.41–4.24
**Information on newspapers or magazines**																
No ®	94.4	2.16	1		1		2.8	1		1		0.63	1		1	
Yes	67.9	10.1	**6.49^c^**	4.9–8.61	1.01	0.70–1.47	16.2	**8.07^c^**	6.18–10.54	1.07	0.73–1.55	5.8	**12.90^c^**	7.98–20.83	1.45	0.88–2.37
**Partner/family smokes**																
No ®	94.5	2.3	1		1		2.6	1		1		0.6	1		1	
Yes	87.8	4.0	**1.86^c^**	1.33–2.61	**0.60^a^**	0.38–0.94	6.2	**2.59^c^**	1.89–3.54	0.67	0.43–1.03	2.0	**3.81^c^**	2.10–6.92	0.67	0.29–1.58
**Partner/family use e-cigarettes**																
No ®	96.3	1.6	1		1		1.7	1		1		0.4	1		1	
Yes	70.5	9.2	**7.79^c^**	5.79–10.5	1.34	0.85–2.12	15.3	**12.32^c^**	9.28–16.34	**1.84^b^**	1.22–2.78	5.0	**18.49^c^**	10.81–31.61	**2.47^a^**	1.18–5.18
**Family use HTPs**																
No ®	96.3	1.6	1		1		1.8	1		1		0.4	1		1	
Yes	48.8	16.1	**20.39^c^**	15.03–27.66	**2.46^c^**	1.54–3.91	26.5	**29.63^c^**	22.49–39.05	**2.90^c^**	1.93–4.37	8.6	**42.29^c^**	25.13–71.16	**2.96^c^**	1.52–5.77
**Friends smoke**																
No ®	95.9	1.7	1		1		2.0	1		1		0.4	1		1	
Yes	88.9	3.8	**2.46^c^**	1.53–3.97	0.62	0.35–1.11	5.6	**3.03^c^**	1.97–4.66	0.60	0.35–1.01	1.7	4.59	1.84–11.45	1.07	0.35–3.28
**Friends use e-cigarettes**																
No ®	97.2	1.2	1		1		1.3	1		1		0.4	1		1	
Yes	77.6	7.5	**7.87^c^**	5.55–11.18	0.75	0.45–1.26	11.5	**11.51^c^**	8.35–15.87	0.92	0.58–1.45	3.4	**11.75^c^**	6.59–20.93	0.62	0.26–1.49
**Friends use HTPs**																
No ®	97.4	1.1	1		1		1.3	1		1		0.3	1		1	
Yes	50.6	16.4	**29.92^c^**	21.73–41.19	**5.78^c^**	3.60–9.30	25.0	**38.08^c^**	27.98–51.81	**4.98^b^**	3.22–7.72	7.9	**55.07^c^**	30.57–99.18	**6.61^c^**	2.91–15.01

* The sample included 6831 observations from 4067 individuals who could contribute more than one observation with follow-up due to the repeated nature of the survey.

† No current HTPs use as the reference, i.e. unaware of HTPs or no HTP use in last month. RRR: relative risk ratio. ARRR: adjusted relative risk ratio.

§ Adjusted by all variables in the table and survey.

® Reference categories. Significant values in bold:

a p<0.05,

b p<0.01,

c p<0.001.

Weekly HTP use (not daily, but at least once/week vs no current HTPs use) was positively associated with: daily smoking (ARRR_daily ≤5 cigarettes vs non-daily_ = 1.96 and ARRR_daily >5 cigarettes vs non-daily_ = 1.88); e-cigarette use (ARRR=6.54); perceived HTPs as less harmful than cigarettes (ARRR=2.25); exposure to HTP information inside and outside tobacco shops where tobacco is sold (ARRR=2.13 and ARRR=2.32, respectively); having a partner or family that uses e-cigarettes (ARRR=1.84); and having family (ARRR=2.90) or friends (ARRR=4.98) that use HTPs. Weekly HTP use was less likely among people with technical and some college studies

(ARRR_technical and some college vs university+_ = 0.49).

Finally, the likelihood of daily HTP use (vs no current HTPs use) was higher among people who: smoked daily (ARRR_daily ≤5 cigarettes vs non-daily_ = 5.53 and ARRR_daily >5 cigarettes vs non-daily_ = 6.46); used e-cigarettes (ARRR=2.59); perceived HTPs as less harmful than cigarettes (ARRR=2.00); were exposed to HTP information inside and outside shops where tobacco is sold (ARRR=3.72 and ARRR=2.44, respectively); having partners and family who use e-cigarettes (ARRR=2.47); and having family (ARRR=2.96) or friends (ARRR=6.61) who use HTPs.

### Smoking quit attempts at follow-up

**Figure 2:**
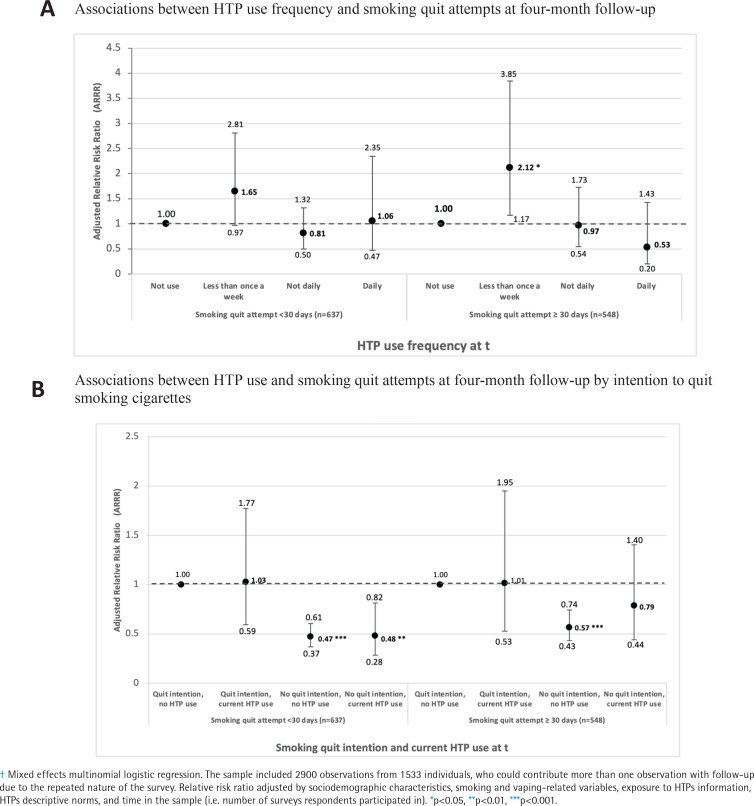
Associations between HTP use and quit attempts at four-month follow-up in an open cohort of Mexican smokers, 2019-2021^†^

In models predicting SQAs at follow-up (Figure 2), monthly HTP use (i.e. less than once a week, but at least once a month) was positively associated with reporting SQAs for at least 30 days (ARRR=2.12), although more frequent HTP use was not associated with SQAs of any duration.

Among those intending to quit smoking, current HTP users were no more likely to report SQAs for either <30 days or ≥30 days compared to non-HTP users. For individuals without quit intentions, both HTP use and non-HTP use were associated with a lower likelihood of SQAs <30 days (ARRR=0.48 and ARRR=0.47, respectively) and ≥30 days only among non-HTP users (ARRR=0.57), when compared to those without quit intentions and who were not using HTPs (Figure 2).

### Changes in smoking frequency

**Figure 3:**
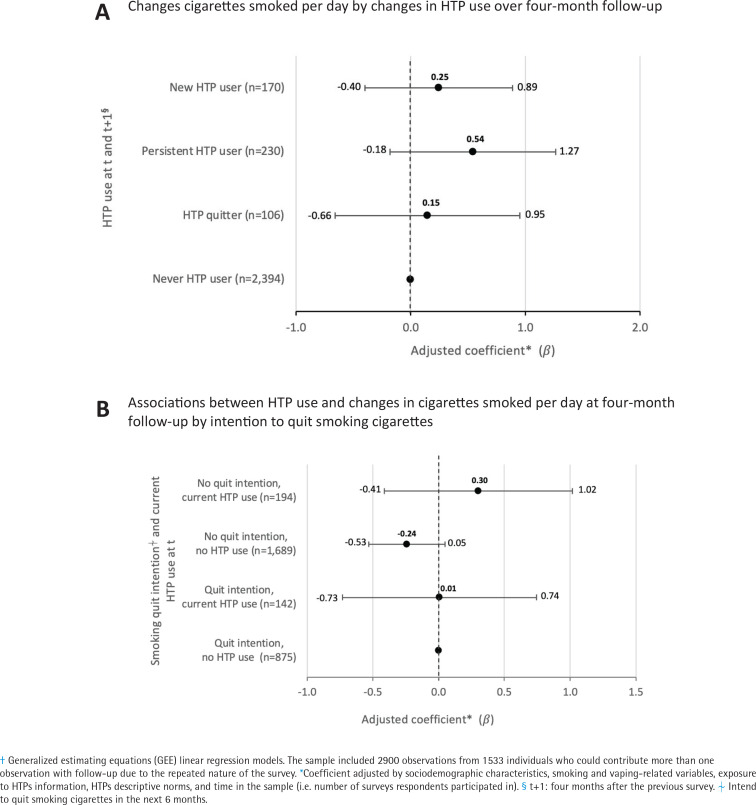
Associations between HTP use and changes in the number of cigarettes smoked per day at four month follow-up in an open cohort of Mexican smokers, 2019-2021^†^

In the GEE linear regression models predicting changes in cigarettes smoked per day, none of the categories of changes in HTPs use between surveys (reference=remain smoker who does not use HTPs) were significantly associated with reducing or increasing cigarettes smoked. Similarly, in the subgroup of people who intended to quit smoking, current HTP use at t (vs no use) was not associated with changes in cigarettes smoked per day(Figure 3).

## DISCUSSION

This study found that Mexican adult smokers were more likely to use HTPs if they smoked daily, perceived HTPs as less harmful than cigarettes, were exposed to HTP-related information, had friends or family members who use HTPs, or if they or their family used e-cigarettes. These results suggest that the adoption of HTPs is shaped by openness to new nicotine technologies – here exemplified by e-cigarettes – among smokers and their personal networks. Furthermore, we found no evidence that HTP use promoted smoking reduction and only limited but unclear evidence on its potential role in cessation (i.e. only among those who used HTPs least frequently). These findings suggest that, contrary to industry claims, the introduction of IQOS, the sole HTP available in the Mexican market, did not contribute to smoking cessation among Mexican smokers^9^.

Similar to prior research^20^, we found that e-cigarette use was an independent correlate of HTP use across all frequencies of use. This tendency toward patterns of poly-tobacco use has been found in diverse populations, including adolescents in Korea and the United States^21,22^. In 2018, 90.5% of current HTP users in high-income Western countries (i.e. Canada, the United States, England, and Australia) smoked cigarettes and used e-cigarettes^23^. Studies in Korea between 2018 and 2019 consistently reported that around 80% to 96.3% of HTP users also smoked cigarettes or ‘triple use’ HTPs, cigarettes, and e-cigarettes^20,24,25^. These emerging patterns of multiple nicotine product use may expose users to high nicotine levels that promote addiction and make it harder for them to quit smoking altogether^24,26^.

Similar to prior research, we found that more frequent smoking was associated with more frequent HTP use. For example, one study found that HTP users who smoke cigarettes reported smoking more cigarettes per day and were more nicotine-dependent than exclusive users of either combustible cigarettes or HTPs^24^. Among Korean populations, the odds of being a former smoker were much lower among dual users of combustible cigarettes and HTPs or e-cigarettes and triple users of HTPs, e-cigarettes, and combustible cigarettes, than among non-HTP and non-e-cigarette users^25^. The lack of association that we found between HTP use and either smoking reduction or quit attempts, suggests that HTP use in Mexico may similarly serve as a complement to rather than a substitute for combustible cigarette use. A prior study involving Mexican smokers indicated that they predominantly use HTPs due to their perceived greater social acceptability compared to combustible cigarettes, as well as their perceived reduced harm to people around them^16^.

In our study, self-reported exposure to HTP information inside and outside venues where tobacco is sold was strongly correlated with the frequency of HTP use. This association is understandable, given promotional efforts by tobacco companies at tobacco outlets that are likely to expose smokers to information about HTPs. However, those who use HTPs are likely to purchase their HTP sticks from venues where HTPs are advertised, thereby potentially challenging causal associations. A study of Japanese HTP users similarly reported high self-reported exposure to HTP advertising in and around stores where tobacco and HTPs are sold^27^. Considering this, further research is needed on the actual content of HTP marketing in countries like Mexico, given that HTPs are often marketed as less harmful and safer alternatives to cigarettes^6^. This could explain our finding that HTP users perceived these products as less harmful in comparison with cigarettes. Analyzing the influences of HTP marketing could offer insights into their potential to promote misperceptions that HTPs aid people to quit smoking^28^, as well as their possible contribution to normalizing tobacco use^29^.

A key finding from our study concerns the strong positive correlations between HTP use and use of HTPs and e-cigarettes among participants’ family members and friends. Similar associations have been found in the UK, where 67.7% of adult HTP users attributed their use to the influence of friends or family members^30^. Also, 45.5% of Korean adult current and former smokers reported initiating HTP use due to family and friends^31^. Social connections, particularly within close circles, play a pivotal role in adopting innovative products like HTPs. The tobacco industry marketing strategy capitalizes on this by including a referral program allowing existing users to receive IQOS product coupons when they advocate for IQOS usage within their circle of family and friends^32^.

The public health impact of HTPs will be determined by several factors, including the effect of HTPs on smoking behaviors, especially successful smoking cessation^2^. In our study, HTP use was unassociated with cessation attempts, except for the counterintuitive finding that smokers who used HTPs least frequently (i.e. <weekly) were more likely than those who did not use HTPs to quit smoking for at least 30 days. In the context of Mexico, where non-daily smoking predominates, low-frequency HTP use could support cessation among infrequent smokers, though longer follow-up is needed to determine relapse rates. Nevertheless, more frequent HTP use would be expected to adequately substitute for cigarette use among the majority of smokers, and our study found no association between more frequent HTP use (i.e. weekly, daily) and quit attempt outcomes. Similarly, a longitudinal study conducted in Hawaii found no association between past-year quit attempts and quitting self-efficacy with the onset of HTP use^33^. Furthermore, in England in 2018, individuals who reported a failed attempt to quit smoking within the past 18 months also had a higher prevalence of HTP use, particularly among those who intended to quit within the next six months or had made a quit attempt over the past 18 months^23^. Hence, our study adds to the growing evidence base that HTP use is not linked to smoking cessation while extending this to include the lack of effects that HTP use appears to have on reducing cigarette smoking frequency.

Intentions to quit smoking are a key precursor to quitting smoking cessation; hence, it is important to evaluate the effects of HTP use among those who intend to quit^2^. While 4.9% of HTP users in our study intended to quit, other studies have also reported relatively lower proportions of smokers who use HTPs and plan to quit smoking; for instance, 4.3% in Korea^10^ and 16.9% in Europe^34^. Moreover, in this last study, only 2% of smokers who intend to quit smoking reported using HTPs as a cessation aid^34^. In our study, individuals who reported both quit intentions and HTP use were no more likely to report SQAs of any duration (i.e. <30 days; ≥30 days) or changes in cigarette smoking frequency compared to those who reported quitting intentions but were not HTP users. These could suggest that some individuals who desire to quit smoking may ultimately abandon their efforts to quit smoking^33^ or consider exploring alternative methods to HTPs^34^. Thus, evaluating changes in the frequency of e-cigarette use or quitting e-cigarettes among both current users and non-users of IQOS could be a future area of research.

### Limitations

The results of this study need to be considered in light of some limitations. Participants were recruited from an online panel used for marketing research, where smokers from lower socioeconomic status groups are underrepresented and where we intentionally over-sampled e-cigarette users. Thus, the sample was not representative of the general population, although this likely increased our ability to sample HTP users, who tend to come from higher SES groups. Another study limitation concerns selection bias, as a significant proportion of participants were lost to follow-up. Participants were more likely to be followed up if they were older and smoked more frequently, both of which may impede smoking cessation. However, other factors associated with greater likelihood of cessation were either positively associated with follow-up (i.e. higher income and education level) or not associated (e.g. dependence, quit attempts, quit intentions). Hence, the directionality of any selection bias effects is difficult to discern and may be negligible. Nevertheless, unmeasured confounders may have biased our results, such as other nicotine tobacco products, although cigarettes and e-cigarettes are by far the most commonly used tobacco products in Mexico. Additionally, our study adjusted for several factors pertinent to smoking cessation, such as individuals’ intention to quit and social influences like peer and family influence. Additionally, smoking cessation was evaluated on SQAs over four months, not long-term abstinence or cessation. Hence, our SQA variable only considered the length of the longest quit attempt, not whether people who tried to quit relapsed, which should be the focus of future research. Nevertheless, a longer duration of quitting attempts predicts successful smoking cessation^35^. Furthermore, we evaluated potential decreases in the number of cigarettes between surveys, yielding results that are generally consistent with those we report for SQAs. Our analyses adjust for smoking frequency in alignment with prevailing usage patterns in Mexico, where most adults who smoke do so less than daily, and even daily smokers^36^ only smoke an average of 5–6 cigarettes per day^18,19^. Different measures of dependence may be needed better explain the persistence of smoking in this population. Although we adjusted for several additional factors pertinent to smoking cessation, such as quit intentions and social influences via peer and family nicotine product use, additional factors may explain the results we found. Lastly, the generalizability of results from this study to other countries may be limited by variations in market dynamics and the availability of alternative HTPs brands distinct from IQOS; however, our findings are likely relevant for other countries in Latin America and similar settings where low smoking frequency predominates.

## CONCLUSIONS

Mexican smokers who use HTPs are likely to be daily smokers and e-cigarette users, with social network members who also use e-cigarettes and HTPs. HTP use was generally not associated with either SQAs or reductions in consumption of cigarettes, with the exception that monthly HTP use predicted SQAs. As such, HTPs do not appear to facilitate smoking cessation in Mexico. Further research is needed to describe better the impact of HTPs on patterns or smoking and e-cigarette use to inform efforts that aim to discourage tobacco use.

## Supplementary Material



## Data Availability

The data supporting this research are available from the authors on reasonable request.
