# Supplementary material for: Heated tobacco product use frequency, smoking quit attempts, and smoking reduction among Mexican adult smokers
Source: Tob Induc Dis. 2024 May 29;22:10.18332/tid/187576. doi: 10.18332/tid/187576 (PMC11135162; doi:10.18332/tid/187576)
Supplement: Supplementary file 1 [file TID-22-90-s1.pdf]

Supplementary file Table 1. Mixed effects multinomial logistic regression[†]of factors associated with HTPs use frequency among smokers from an open cohort study in Mexico, 20019-2021*

	**Do not current use (n=4,925)**	**Less than once a week, but at least once a month (n=176)**					**Not daily but at least once a week (n=264)**					**Daily (n=82)**				
	**%**	**%**	**RRR**	**(95%CI)**	**ARRR**[§]	**(95%CI)**	**%**	**RRR**	**(95%CI)**	**ARRR**[§]	**(95%CI)**	**%**	**RRR**	**(95%CI)**	**ARRR**[§]	**(95%CI)**
**Sex**																
Female	91.2	3.1	Ref		Ref		4.5	Ref		Ref		1.3	Ref		Ref	
Male	89.7	3.4	1.13	(0.80, 1.60)	1.06	(0.73-1.55)	5.2	1.41	(0.92-1.69)	1.01	(0.68-1.48)	1.7	1.427	(0.88-2.31)	1.03	(0.58-1.83)
**Age**																
18-29	88.3	4.5	Ref		Ref		5.5	Ref		Ref		1.8	Ref		Ref	
30-39	87.4	4.2	0.96	(0.66-1.41)	0.72	(0.46-1.14)	6.0	1.11	(0.75-1.62)	0.7	(0.43-1.14)	2.5	1.41	(0.80-2.51)	0.71	(0.37-1.33)
40-49	92.1	2.2	**0.47**[1]	(0.28-0.79)	**0.49**[1]	(0.26-0.91)	4.8	0.84	(0.52-1.37)	0.8	(0.44-1.45)	0.9	0.51	(0.24-1.09)	0.43	(0.18-1.03)
50+	95.7	1.3	**0.27**[3]	(0.15-0.49)	**0.48**[1]	(0.25-0.94)	2.6	**0.44**[2]	(0.26-0.75)	0.92	(0.47-1.77)	0.4	**0.22**[1]	(0.08-0.57)	0.37	(0.13-1.10)
**Education**																
High school and less	93.6	2.3	**0.39**[3]	(0.27-0.57)	0.72	(0.45-1.17)	3.5	**0.33**[3]	(0.23-0.48)	0.7	(0.44-1.12)	0.6	**0.17**[3]	(0.08-0.38)	**0.36**[1]	(0.14-0.93)
Technical and some college	95.3	2.3	**0.38**[3]	(0.25-0.57)	0.86	(0.53-1.39)	1.6	**0.16**[3]	(0.10-0.44)	**0.41**[2]	(0.24-0.71)	0.9	**0.24**[3]	(0.13-0.44)	0.80	(0.38-1.70)
University and more	82.6	5.2	Ref		Ref		9.2	Ref		Ref		3.1	Ref		Ref	
**Household income**																
Less than 8,000MXN	93.8	2.3	Ref		Ref		2.9	Ref		Ref		1.1	Ref		Ref	
8,001 to 15,000MXN	91.3	3.7	**1.68**[1]	(1.04-2.69)	1.15	(0.64-2.05)	4.1	1.46	(0.91-2.35)	0.95	(0.54-1.69)	1.0	0.91	(0.47-1.77)	0.44	(0.19-1.04)
15,001+ MXN	86.6	3.6	**1.72**[1]	(1.08-2.72)	1.07	0.61-1.89)	6.8	**2.55**[3]	(1.61-4.03)	1.33	(0.71-2.49)	2.2	**2.18**[1]	(1.15-4.15)	0.91	(0.39-2.13)
I don't know	97.3	1.1	0.48	(0.14-1.59)	1.64	(0.54-5.03)	1.5	0.51	(0.17-1.47)	1.94	(0.71-5.27)	0.4	0.33	(0.04-2.09)	1.58	(0.11-22.38)
**Smoking frequency**																
Non-daily	93.6	2.8	Ref		Ref		3.2	Ref		Ref		0.4	Ref		Ref	
Daily <=5 cigarettes	89.6	2.9	1.07	(0.69-1.67)	1.2	(0.72-2.01)	5.4	**1.77**[2]	(1.21-2.59)	**1.86**[2]	(1.17-2.95)	2.1	**5.55**[3]	(2.63-11.71)	**5.93**[3]	(2.59-13.60)
Daily >5 cigarettes	85.1	4.3	**1.65**[2]	(1.14-2.38)	**1.86**[1]	(1.13-3.06)	7.5	**2.59**[3]	(1.81-3.71)	**2.35**[3]	(1.50-3.68)	3.1	**8.73**[3]	(4.39-17.39)	**8.57**[3]	(3.90-18.83)
**E-cigarette use**																
No	98.3	0.6	Ref		Ref		0.7	Ref		Ref		0.4	Ref		Ref	
Yes	74.7	8.4	**17.47**[3]	(11.17-27.32)	**6.31**[3]	(3.60-11.05)	13.1	**24.04**[3]	(15.57-37.12)	**5.78**[3]	(3.37-9.93)	3.7	**12.76**[3]	(7.08-22.99)	**2.27**[1]	(1.05-4.91)
**Recent smoking quit attempt**																
No	92.3	2.6	Ref		Ref		3.7	Ref		Ref		1.3	Ref		Ref	
Yes	87.5	4.2	**1.71**[2]	(1.24-2.35)	**1.64**[1]	(1.07-2.50)	6.6	**1.86**[3]	(1.38-2.50)	1.24	(0.83-1.85)	1.4	1.38	(0.89-2.14)	1.02	(0.53-1.97)
**Intention to quit smoking**																
No	91.3	3.4	Ref		Ref		4.1	Ref		Ref		1.3	Ref		Ref	
In the next six months	88.9	3.0	0.92	(0.67-1.27)	0.6	(0.39-0.91)	6.2	**1.57**[2]	(1.18-2.08)	1.26	(0.85-1.89)	1.8	1.43	(0.91-2.24)	1.23	(0.65-2.32)
**Smoking dependency (WISDOM) (mean, SD)**	2.7 (0.9)	3.04 (0.8)	**1.46**[3]	(1.27-1.67)	0.88	(0.72-1.09)	3.18 (0.8)	**1.72**[3]	(1.49-1.99)	0.95	(0.75-1.19)	3.28 (0.9)	**1.92**[3]	(1.45-2.55)	0.96	(0.70-1.31)
**Low relative risk perception of HTPs compared to combustible cigarettes**																
No	94.8	1.7	Ref		Ref		2.6	Ref		Ref		0.9	Ref		Ref	
Yes	66.5	11.3	**9.26**[3]	(6.68-12.84)	**2.71**[3]	(1.82-4.05)	17.2	**9.55**[3]	(7.18-12.70)	**2.56**[3]	(1.78-3.39)	5.1	**8.51**[3]	(5.42-13.35)	**2.15**[2]	(1.27-3.63)
**Inside shops/ stores that sell tobacco**																
No	95.1	1.8	Ref		Ref		2.6	Ref		Ref		0.5	Ref		Ref	
Yes	57.0	13.1	**11.89**[3]	(8.49-16.67)	**2.31**[3]	(1.47-3.63)	20.9	**13.43**[3]	(10.13-17.80)	**2.12**[3]	(1.42-3.18)	9.0	**32.43**[3]	(18.77-56.04)	**4.33**[3]	(2.43-7.72)
**Outside shops/stores that sell tobacco**																
No	95.9	1.6	Ref		Ref		2.0	Ref		Ref		0.5	Ref		Ref	
Yes	63.3	11.4	**10.99**[3]	(7.82-15.45)	**2.20**[2]	(1.39-3.47)	18.8	**14.13**[3]	(10.61-18.82)	**2.63**[3]	(1.81-3.83)	6.5	**20.27**[3]	(11.69-35.14)	**2.78**[2]	(1.51-5.14)
**Information on newspapers or magazines**																
No	94.8	1.96	Ref		Ref		2.72	Ref		Ref		0.57	Ref		Ref	
Yes	67.3	10.0	**7.19**[3]	(5.21-9.91)	1.13	(0.74-1.72)	16.2	**8.36**[3]	(6.25-11.18)	1.07	(0.70-1.63)	6.5	**16.2**[3]	(9.72-27.01)	**1.72**[1]	(1.00-2.97)

**Partner/family smokes**																
No	94.6	2.2	Ref		Ref		2.6	Ref		Ref		0.6	Ref		Ref	
Yes	88.1	3.8	**1.86**[2]	(1.29-2.70)	**0.60**[1]	(0.37-0.98)	6.1	**2.56**[3]	(1.79-3.68)	0.66	(0.41-1.07)	2.0	**3.41**[3]	(1.82-6.39)	0.50	(0.20-1.26)
**Partner/family use e-cigarette**																
No	96.4	1.6	Ref		Ref		1.7	Ref		Ref		0.4	Ref		Ref	
Yes	70.7	8.8	**7.67**[3]	(5.50-10.69)	1.32	(0.80-2.18)	15.3	**12.45**[3]	(9.01-17.20)	**1.84**[2]	(1.13-2.99)	5.3	**20.06**[3]	(11.12-36.18)	**2.45**[1]	(1.03-5.85)
**Family use HTPs**																
No	96.4	1.5	Ref		Ref		1.7	Ref		Ref		0.4	Ref		Ref	
Yes	48.1	15.6	**21.00**[3]	(15.04-29.33)	**2.26**[2]	(1.31-3.89)	26.9	**30.97**[3]	(22.83-42.00)	**2.80**[3]	(1.79-4.38)	9.5	**50.49**[3]	(28.93-88.11)	**3.60**[2]	(1.61-8.06)
**Friends smoke**																
No	96.4	1.5	Ref		Ref		1.7	Ref		Ref		0.4	Ref		Ref	
Yes	89.1	3.6	**2.59**[2]	(1.46-4.58)	0.79	(0.39-1.59)	5.5	**3.50**[3]	(2.07-5.90)	0.83	(0.44-1.57)	1.8	**4.70**[2]	(1.70-12.98)	1.40	(0.42-4.69)
**Friends use e-cigarette**																
No	97.3	1.2	Ref		Ref		1.2	Ref		Ref		0.3	Ref		Ref	
Yes	77.4	7.1	**7.56**[3]	(5.21-10.96)	0.7	(0.41-1.12)	11.8	**12.88**[3]	(8.95-18.54)	1.05	(0.62-1.78)	3.7	**13.82**[3]	(7.37-25.92)	0.65	(0.24-1.75)
**Friends use HTPs**																
No	97.5	1.0	Ref		Ref		1.2	Ref		Ref		0.3	Ref		Ref	
Yes	49.4	16.2	**32.23**[3]	(22.30-45.59)	**5.81**[3]	(3.27-10.33)	25.8	**41.42**[3]	(29.35-58.45)	**4.57**[3]	(2.83-739)	8.7	**66.53**[3]	(34.96-126.62)	**6.24**[3]	(2.44-15.95)

*The sample included 6831 observations from 4,067 individuals, who could contribute more than one observation with follow-up due to the repeated nature of the survey.

†No current HTPs use as the reference (i.e., unaware of HTPs or no HTP use in last month)

RRR (Relative Risk Ratio) and ARRR (Adjusted Relative Risk Ratio)

§Adjusted by all variables in the table and survey

Significant values in bold: 1 p-value, $p < 0.05$; 2 p-value, $p < 0.01$; 3 p-value, $p < 0.001$.

Supplementary file Table 2. Mixed-effects logistic regression of factors associated with being followed in a consecutive survey among smokers from an open cohort study in Mexico, 2019-2021*

	No follow-up (n=2,544)	Follow-up (n=2,900)				
	**%**	**%**	**OR**	**(95%CI)**	**AOR**	**(95%CI)**
**Sex**						
Female	47.5	46.5	Ref		Ref	
Male	52.2	53.5	1.02	(0.88-1.19)	0.88	(0.73-1.07)
**Age**						
18-29	34.9	20.6	Ref		Ref	
30-39	31.2	30.4	**1.89**[3]	(1.55-2.29)	**2.15**[3]	(1.66-2.77)
40-49	16.2	22.4	**2.86**[3]	(2.28-3.58)	**3.89**[3]	(2.87-5.26)
50+	17.7	26.6	**3.3**[3]	(2.64-4.11)	**5.08**[3]	(3.69-7.00)
**Education**						
High school and less	37.2	35.5	**0.69**[3]	(0.57-0.82)	0.83	(0.65-1.06)
Technical and some college	33.5	27.2	**0.58**[3]	(0.48-0.70)	**0.65**[3]	(0.51-0.83)
University and more	28.3	35.5	Ref		Ref	
**Household income**						
Less than 8,000MXN	25.8	18.4	Ref		Ref	
8,001 to 15,000MXN	29.7	30.7	**1.58**[3]	(1.29-1.93)	**1.48**[2]	(1.16-1.89)
15,001+ MXN	39.9	45.7	**1.84**[3]	(1.51-2.23)	**1.38**[1]	(1.07-1.78)
I don't know	4.6	5.1	**1.75**[2]	(1.21-2.52)	**1.68**[1]	(1.08-2.61)
**Smoking frequency**						
Non-daily	54.1	48.4	Ref		Ref	
Daily <=5 cigarettes	21.3	23.9	**1.24**[1]	(1.04-1.48)	0.99	(0.79-1.24)
Daily >5 cigarettes	24.7	27.7	**1.23**[1]	(1.03-1.47)	**0.76**[1]	(0.60-0.97)
**E-cigarette use**						
No	68.6	64.9	Ref		Ref	
Yes	31.4	35.1	**1.19**[1]	(1.02-1.38)	**1.43**[2]	(1.13-1.81)
**Recent smoking quit attempt**						
No	59.2	61.0	Ref		Ref	
Yes	40.8	39.0	0.93	(0.80-1.07)	0.95	(0.79-1.16)
**Intention to quit smoking**						
No	63.0	64.9	Ref		Ref	
In the next six months	37.0	35.1	0.93	(0.80-1.07)	0.93	(0.77-1.13)
**Smoking dependency (WISDOM) (mean, SD)**	2.70 (0.9)	2.78 (0.9)	**1.12**[2]	(1.03-1.21)	1.09	(0.98-1.21)
**Current HTP use**						
No	92.7	88.4	Ref		Ref	
Yes	7.3	11.6	**1.62**[3]	(1.26-2.08)	1.45	(1.01-2.10)
**Low relative risk perception of HTPs compared to combustible cigarettes**						
No	86.1	83.0	Ref		Ref	
Yes	14.0	17.1	1.21	(1.00-1.47)	1.07	(0.84-1.38)
**Inside shops/ stores that sell tobacco**						
No	88.9	86.7	Ref		Ref	
Yes	11.1	13.3	1.16	(0.94-1.44)	0.92	(0.68-1.24)
**Outside shops/stores that sell tobacco**						
No	84.1	81.3	Ref		Ref	
Yes	14.9	18.7	**1.28**[1]	(1.06-1.55)	1.10	(0.84-1.45)
**Information on newspapers or magazines**						
No	86.1	82.64	Ref		Ref	
Yes	14.0	17.4	**1.23**[1]	(1.01-1.49)	0.98	(0.75-1.28)
**Partner/family smokes**						
No	33.3	36.9	Ref		Ref	
Yes	66.7	63.1	**0.83**[1]	(0.72-0.97)	0.89	(0.74-1.08)
**Partner/family use e-cigarette**						
No	76.2	77.2	Ref		Ref	
Yes	23.8	22.8	0.9	(0.76-1.06)	**0.73**[1]	(0.56-0.93)
**Family use HTPs**						
No	89.3	86.2	Ref		Ref	
Yes	10.7	13.8	**1.34**[2]	(1.08-1.67)	1.31	(0.92-1.86)
**Friends smoke**						
No	17.9	18.5	Ref		Ref	
Yes	82.1	81.5	0.94	(0.78-1.13)	0.87	(0.70-1.10)
**Friends use e-cigarette**						
No	65.1	65.5	Ref		Ref	
Yes	34.9	34.5	0.91	(0.81-1.10)	0.88	(0.70-1.11)
**Friends use HTPs**						

No	87.6	83.2	Ref		Ref	
Yes	12.4	16.8	1.35[2]	(1.10-1.66)	1.19	(0.83-1.69)
**Wave**						
4	23.2	27.5	Ref		Ref	
5	23.8	25.7	**0.48**[3]	(0.37-0.62)	**0.56**[3]	(0.44-0.72)
6	23.7	25.1	**0.35**[3]	(0.26-0.49)	**0.44**[3]	(0.33-0.58)
7	29.3	21.7	**0.16**[3]	(0.11-0.24)	**0.20**[3]	(0.14-0.28)

*The sample included 5,447 observations from 3,294 individuals, who could contribute more than one observation with follow-up due to the repeated nature of the survey.

OR (Odds Ratio), AOR (Adjusted Odds Ratio)

§Adjusted by all variables in the table and survey

Significant values in bold: 1 p-value, $p < 0.05$; 2 p-value, $p < 0.01$; 3 p-value, $p < 0.001$.

© 2024 Cruz-Jiménez L. et al.
